# Longitudinal Associations of Clinical and Biochemical Head Injury Biomarkers With Head Impact Exposure in Adolescent Football Players

**DOI:** 10.1001/jamanetworkopen.2023.16601

**Published:** 2023-05-30

**Authors:** Taylor R. Zuidema, Jeffrey J. Bazarian, Kyle A. Kercher, Rebekah Mannix, Reuben H. Kraft, Sharlene D. Newman, Keisuke Ejima, Devin J. Rettke, Jonathan T. Macy, Jesse A. Steinfeldt, Keisuke Kawata

**Affiliations:** 1 Department of Kinesiology, Indiana University School of Public Health–Bloomington; 2 Program in Neuroscience, College of Arts and Sciences, Indiana University, Bloomington; 3 Department of Emergency Medicine, University of Rochester School of Medicine and Dentistry, Rochester, New York; 4 Department of Medicine, Division of Emergency Medicine, Boston Children’s Hospital, Harvard Medical School, Boston, Massachusetts; 5 Department of Mechanical and Biomedical Engineering, Pennsylvania State University, University Park; 6 Institute of Computational and Data Sciences, Pennsylvania State University, University Park; 7 Alabama Life Research Institute, University of Alabama, Tuscaloosa; 8 Lee Kong Chian School of Medicine, Nanyang Technological University, Singapore; 9 Department of Applied Health Science, Indiana University School of Public Health–Bloomington; 10 Department of Counseling and Educational Psychology, School of Education, Indiana University, Bloomington

## Abstract

**Importance:**

Consequences of subconcussive head impacts have been recognized, yet most studies to date have included small samples from a single site, used a unimodal approach, and lacked repeated testing.

**Objective:**

To examine time-course changes in clinical (near point of convergence [NPC]) and brain-injury blood biomarkers (glial fibrillary acidic protein [GFAP], ubiquitin C-terminal hydrolase-L1 [UCH-L1], and neurofilament light [NF-L]) in adolescent football players and to test whether changes in the outcomes were associated with playing position, impact kinematics, and/or brain tissue strain.

**Design, Setting, and Participants:**

This multisite, prospective cohort study included male high school football players aged 13 to 18 years at 4 high schools in the Midwest during the 2021 high school football season (preseason [July] and August 2 to November 19).

**Exposure:**

A single football season.

**Main Outcomes and Measures:**

The main outcomes were NPC (a clinical oculomotor test) and serum levels of GFAP, UCH-L1, and NF-L. Participants’ head impact exposure (frequency and peak linear and rotational accelerations) was tracked using instrumented mouthguards, and maximum principal strain was computed to reflect brain tissue strain. Players’ neurological function was assessed at 5 time points (preseason, post–training camp, 2 in season, and postseason).

**Results:**

Ninety-nine male players contributed to the time-course analysis (mean [SD] age, 15.8 [1.1] years), but data from 6 players (6.1%) were excluded from the association analysis due to issues related to mouthguards. Thus, 93 players yielded 9498 head impacts in a season (mean [SD], 102 [113] impacts per player). There were time-course elevations in NPC and GFAP, UCH-L1, and NF-L levels. Compared with baseline, the NPC exhibited a significant elevation over time and peaked at postseason (2.21 cm; 95% CI, 1.80-2.63 cm; *P* < .001). Levels of GFAP and UCH-L1 increased by 25.6 pg/mL (95% CI, 17.6-33.6 pg/mL; *P* < .001) and 188.5 pg/mL (95% CI, 145.6-231.4 pg/mL; *P* < .001), respectively, later in the season. Levels of NF-L were elevated after the training camp (0.78 pg/mL; 95% CI, 0.14-1.41 pg/mL; *P* = .011) and midseason (0.55 pg/mL; 95% CI, 0.13-0.99 pg/mL; *P* = .006) but normalized by the end of the season. Changes in UCH-L1 levels were associated with maximum principal strain later in the season (0.052 pg/mL; 95% CI, 0.015-0.088 pg/mL; *P* = .007) and postseason (0.069 pg/mL; 95% CI, 0.031-0.106 pg/mL; *P* < .001).

**Conclusions and Relevance:**

The study data suggest that adolescent football players exhibited impairments in oculomotor function and elevations in blood biomarker levels associated with astrocyte activation and neuronal injury throughout a season. Several years of follow-up are needed to examine the long-term effects of subconcussive head impacts in adolescent football players.

## Key Points

**Question:**

Are playing position, impact kinematics, and/or brain tissue strain associated with longitudinal changes in blood biomarkers and neuro-ophthalmologic functions in adolescent football players?

**Findings:**

In this cohort study of 99 adolescent football players, blood biomarker levels (glial fibrillary acidic protein, ubiquitin C-terminal hydrolase-L1 [UCH-L1], and neurofilament light) and the near point of convergence increased throughout the season. All playing positions showed similar degrees of elevations, and UCH-L1 changes were associated with brain tissue strain and head impact kinematics.

**Meaning:**

In this study, football players had oculomotor impairments and changes in blood biomarkers related to cellular injury, and some of these changes were associated with repetitive head impacts.

## Teaser

This cohort study examines associations of brain injury–related clinical and blood biomarkers with exposure to head impacts among adolescent football players during a football season.

## Introduction

Repetitive subconcussive head impacts in sports have gained the spotlight in the field of neurology due to their potentially insidious, long-term effects on the brain.^1,2^ Because these head impacts are often asymptomatic, many contact-sport athletes sustain hundreds of head impacts in a single season.^3^ Yet, it remains uncertain whether there is a limit of tolerance to subconcussive head impacts and whether neurological effects of head impacts are dose- and intensity-dependent.^4,5,6^

The quest to explore highly sensitive measures to inspect brain health has yielded several candidate biomarkers, such as clinical oculomotor testing (near point of convergence [NPC]) and brain-derived blood biomarkers, including glial fibrillary acidic protein (GFAP), ubiquitin C-terminal hydrolase-L1 (UCH-L1), and neurofilament light (NF-L). These measures have been shown to elevate in concert with head impact exposure during acute and subacute phases,^7,8,9,10,11,12,13^ suggesting that NPC is one of the most sensitive clinical tools to detect subconcussive brain injury,^14^ whereas GFAP, UCH-L1, and NF-L are useful to gauge the severity of microstructural injury and inflammatory responses after head impacts.^15,16^

Despite a decade of effort, research in subconcussive brain injury remains inconclusive due to several limitations, ranging from a small-scale, single-site study with a limited number of time points^17^ to head impact sensors not reflecting strains in the brain tissue, which is a missing component when studying the associations between biomechanical forces and neurobiological responses.^18^ Finite element modeling can estimate the extent of brain tissue strain, as described by maximum principal strain (MPS).^19,20^ The recent evolution in machine learning and computational modeling techniques has begun suggesting that strain-derived metrics may better capture the brain tissue deformation upon head impacts than do head or skull acceleration metrics like peak linear acceleration (PLA) and peak rotational acceleration (PRA).^21^ However, to our knowledge, MPS has not been studied in the context of repetitive subconcussive head impacts.

We conducted a prospective, multisite, longitudinal study to evaluate time-course neurological responses in 1 season of high school football. We further explored the associations of players’ position, head impact kinematics (frequency, PLA, and PRA), and strain metrics (MPS) with time-course changes in NPC and GFAP, UCH-L1, and NF-L levels. Our hypothesis was that there would be significant elevations in NPC and levels of all 3 blood biomarkers over time, by which the elevations would intensify later in the season but show slight recovery at postseason. Time-course changes in the outcomes were further explored in relation to players’ position, head impact kinematics, and brain tissue strains to identify factors associated with brain response to subconcussive head impacts.

## Methods

### Participants

This multisite cohort study included 99 male high school football players from 4 high schools in the Midwest. The study was conducted during the 2021 preseason (July) for baseline assessment, as well as throughout the 2021 football season, from August 2 to November 19. Inclusion criteria were being a current member of the high school football team and aged 13 to 18 years. All participants and their legal guardians provided informed consent online, and the Indiana University institutional review board approved the study protocol. This study followed the Strengthening the Reporting of Observational Studies in Epidemiology (STROBE) reporting guideline.

### Study Procedures

Data collection took place during preseason in July (T1 [baseline]), after training camp in August (T2), at 2 in-season time points (September [T3] and October [T4]), and postseason in November or December (T5). The training camp, which consisted of a high-intensity training period to prepare for the upcoming season, took place from the start day of the season (August 2) until the first game later in August. For T2, T3, and T4, there was an interval of at least 24 hours between the last contact practice and data collection. For T5, there was an interval of at least 14 days since the final game (eFigure in Supplement 1). Data collection was conducted in a large group setting at each school, and blood samples and NPC were collected at each time point. During preseason data collection, all participants were fitted with an Impact Monitor Mouthguard (Prevent Biometrics, Inc) to measure head impact kinematics from every practice and game. For the exploratory analysis, based on playing positions, participants were categorized as linemen (offensive and defensive), hybrid (tight ends, running backs, and linebackers), or skill groups (wide receivers, cornerbacks, safeties, and quarterbacks). Race and ethnicity data were ascertained by participant report; race categories were American Indian or Alaska Native, Asian, Black/African American, Native Hawaiian or Pacific Islander, White, or multiracial, and ethnicity categories were Latino/Hispanic or not Latino/Hispanic.

### Head Impact Measurement

The Impact Monitor Mouthguard incorporates data from a triaxial accelerometer (ADXL372) and gyroscope (BMG250) to provide 6-*df* spatial and temporal estimates of linear and rotational head accelerations during impact. When an axis of acceleration exceeds a preset threshold of 10*g*, an impact event triggers data collection. The sampling rate is 3.2 kHz, and impact data are collected for 50 milliseconds.^22,23^ Cumulative frequency, PLA, and PRA were used in our analyses.

### Video Validation of Head Impact Measures

The film analysis was conducted by research assistants who were blinded to research outcomes. Interrater reliability was assessed and resulted in excellent reliability, with an intraclass correlation coefficient (ICC) of 0.96 (95% CI, 0.94- 0.99; *P* < .001). Randomly stratified head impact data (1785 impacts) derived from both practices and games were assessed and classified as either a true-positive or false-positive impact. An impact could be to an athlete’s head or body, because both can induce a head acceleration event.^24^ Positive predictive values were then computed.

### Maximum Principal Strain

We used the Brain Simulation Research Platform,^25^ established by 1 of the authors (R.H.K.), to estimate brain tissue strain, as reflected in MPS, using the finite element method. The code for a finite element model was validated against widely accepted codes, such as Abaqus and LS-DYNA, and resulted in tight coupling for tension, compression, and shear simulations. The detailed coding and processing were described previously.^26^ Maximum principal strain is computed by solving the equation of motion dynamically using the explicit dynamic finite element method,^19^ which incorporates the combination of linear and rotational accelerations from 6 axes. The Green-Lagrangian strain^26^ is computed for each element. Once the strain value is found, the MPS value is the largest eigenvalue of the strain matrix. The highest MPS value over both time and elements represents the maximum strain experienced by the brain during the head impact. Cumulative MPS and the number of head impacts with MPS of 10 or greater, which is classified as medium or greater strain, were used in our analyses.

### NPC

The NPC was assessed based on our established protocol.^7,8,9^ In brief, a target was moved down the length of the accommodation ruler toward the participant’s eyes. The NPC was taken when eye misalignment was observed by the tester or when the participant verbally signaled once they experienced diplopia. The assessment was repeated twice, and the mean NPC was then used for analyses. Three examiners (T.R.Z., K.A.K., and D.J.R.) who were blinded to head impact exposure had excellent intrarater (ICC, 0.94; 95% CI, 0.89-0.95) and interrater reliabilities (ICC, 0.90; 95% CI, 0.83-0.94).

### Blood Sample Obtainment and Assays

At each time point, 1.2 mL of capillary blood was collected from the upper arm using a noninvasive Tasso serum sampling kit (Tasso Inc). Serum was separated by centrifugation and stored at −80 °C until analysis. The GFAP, UCH-L1, and NF-L measurements were performed using the Human Neurology 4-Plex A assay on a Quanterix SR-X system. During the validation processes, GFAP, UCH-L1, and NF-L resulted in nearly identical expressions (coefficient of variation, <2.0) between venous blood samples and capillary blood samples via the Tasso device. However, tau levels in the capillary samples were consistently higher (approximately 2- to 10-fold) than in the venous samples; thus, tau was not included in the study. The same board-certified personnel (T.R.Z.), who was blinded to players’ positions and head impact kinematics, performed all assays. The mean (SD) intraassay coefficients of variation for the samples were 8.3% (6.0%) for NF-L, 3.7% (2.7%) for GFAP, and 8.2% (7.1%) for UCH-L1. The lowest detection limit of the assay was 0.024 pg/mL for NF-L, 0.221 pg/mL for GFAP, and 1.740 pg/mL for UCH-L1. All samples from each participant were assayed on the same 96-well plate.

### Statistical Analysis

To examine the time-course changes in neurological outcomes, we conducted a series of multivariable mixed-effects regression models (MMRMs) on the primary outcomes (NPC and GFAP, UCH-L1, and NF-L levels). The first MMRM evaluated the time effects by analyzing the degrees of changes in outcomes over time relative to baseline (T1). Given that we used 1 MMRM per outcome (n = 4), we considered 2-sided *P* < .01 as statistically significant. The second MMRM focused on group differences to assess whether the trajectory of time-course changes differed among linemen, hybrid, and skill groups. A subclass of MMRM was used to account for both individual differences at baseline and over time to estimate the effects of group (linemen, hybrid, and skill), time (T1 to T5), and group-by-time interactions. Age, number of previous concussions, and years of tackle football experience were included as covariates in these time-course analyses.

Linear regression models were used to examine whether the changes in outcome variables at each time point were associated with cumulative subconcussive head impacts. Head impact kinematic (frequency, PLA, and PRA) and simulation (sum of MPS and MPS ≥10) data up to each time point were organized, and changes in neurological outcomes were regressed against impact metrics (factors). The significance level was set at 2-sided *P* < .01 to reflect 1 regression model per factor. All analyses were conducted using R, version 3.4.1 (R Project for Statistical Computing) with the package nlme.

## Results

### Demographics and Head Impact Kinematics

**Figure 1.**
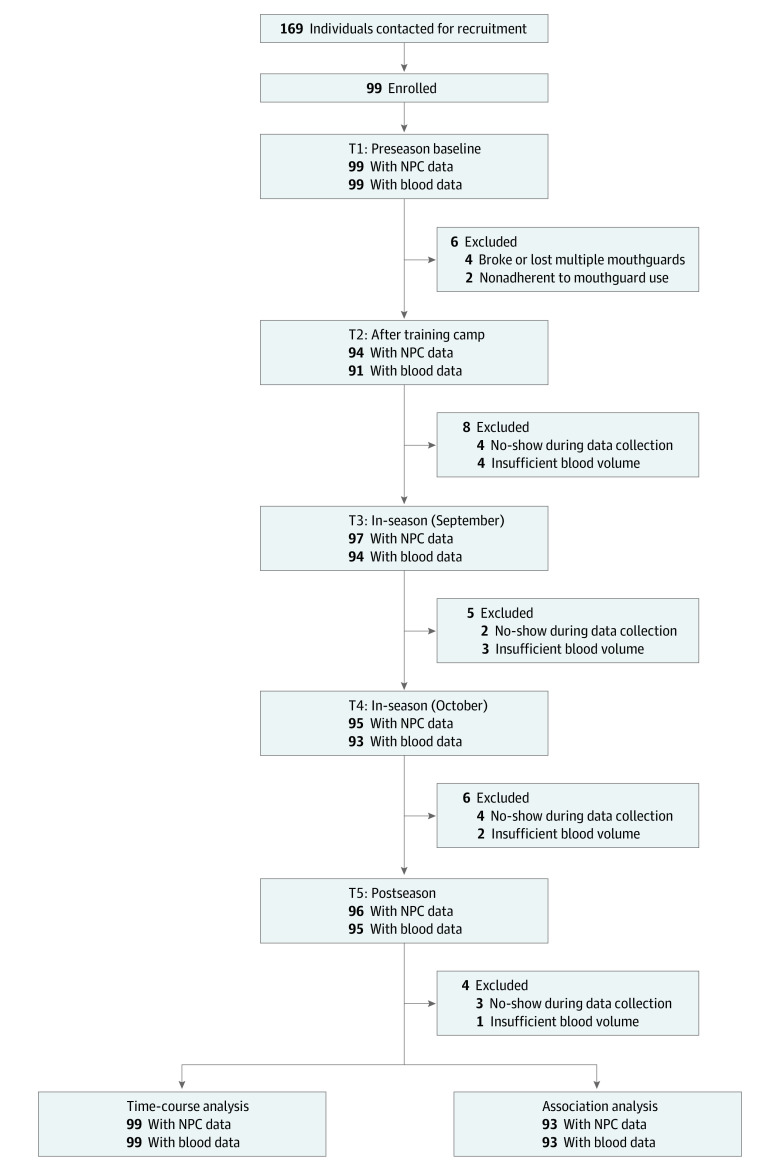
Study Flowchart NPC indicates near point of convergence.

Ninety-nine high school football players were initially enrolled in the study. Data from these 99 players (mean [SD] age, 15.8 [1.1] years) were used for the primary analysis on the time-course changes in neurological outcomes. The sample consisted of all males. None of the players were American Indian, Alaska Native, Asian, Native Hawaiian, or Pacific Islander; 9 (9.1%) were Black/African American; 87 (87.9%) were White; and 3 (3.0%) were multiracial. A total of 6 players (6.1%) were Latino/Hispanic, and 93 (93.9%) not Latino/Hispanic. Ninety-three players yielded 9498 head impacts in the season (mean [SD], 102 [113] impacts per player). Demographics and head impact kinematics are summarized in the Table. Data from 6 players (6.1%) were excluded from the association analysis due to lack of head impact data (eg, mouthguard adherence, breakage). There were missing samples at each follow-up time point (T2 to T5) due to either no-show during data collection (4 at T2, 2 at T3, 4 at T4, and 3 at T5) or insufficient blood volume (4 at T2, 3 at T3, 2 at T4, and 1 at T5). Figure 1 shows the study flow.

**Table.** Group Demographics and Head Impact Kinematics

Variable	Players^a^			
	Overall (N = 99)	Linemen (n = 38)	Hybrid (n = 31)	Skill (n = 30)
Age, mean (SD), y	15.8 (1.1)	15.9 (1.1)	15.8 (1.1)	15.6 (1.1)
BMI, mean (SD)	26.2 (5.5)	31.6 (6.3)	24.9 (3.1)	22.0 (2.2)
Previous concussions, No.				
0	83 (83.9)	33 (86.6)	27 (87.1)	23 (76.7)
1	13 (13.1)	5 (13.4)	4 (12.9)	4 (13.3)
2	3 (3.0)	0	0	3 (10.0)
Years of tackle football experience, mean (SD)	5.1 (2.9)	5.0 (3.0)	6.0 (2.9)	4.0 (2.6)
Race				
American Indian or Alaska Native	0	0	0	0
Asian	0	0	0	0
Black/African American	9 (9.1)	3 (7.9)	1 (3.2)	5 (16.7)
Native Hawaiian or Pacific Islander	0	0	0	0
White	87 (87.9)	34 (89.5)	29 (93.6)	24 (80.0)
Multiracial	3 (3.0)	1 (2.6)	1 (3.2)	1 (3.3)
Ethnicity				
Latino/Hispanic	6 (6.1)	3 (7.9)	1 (3.2)	2 (6.7)
Not Latino/Hispanic	93 (93.9)	35 (92.1)	30 (96.8)	28 (93.3)
Impact kinematics for season, mean (SD)				
Cumulative impact count	102 (113)	115 (124)	112 (93)	74 (115)
Cumulative PLA, *g*	1641.6 (1856.2)	1748.5 (1915.6)	1899.3 (1632.2)	1244.3 (2069.7)
Cumulative PRA, krad/s^2^	32.5 (39.4)	35.4 (42.9)	34.2 (31.5)	27.5 (45.0)
MPS for season, mean (SD)				
Sum of MPS per player	816.05 (926.71)	826.91 (895.66)	973.03 (876.45)	646.71 (1009.99)
Hits ≥10 MPS per player, No.	22.89 (29.29)	17.91 (21.54)	30.62 (32.24)	20.83 (33.06)

Abbreviations: BMI, body mass index (calculated as weight in kilograms divided by height in meters squared); MPS, maximum principal strain; PLA, peak linear acceleration; PRA, peak rotational acceleration.

^a^ Data are presented as the number (percentage) of players unless otherwise indicated.

### Video Validation of Head Impacts

Of the 1785 head impacts that were selected to be reviewed for video validation, 1670 (93.6%) were visually confirmed while 115 (6.4%) were not. This equates to a positive predictive value of 93.6%.

### Time-Course Changes in NPC and Blood Biomarker Levels

**Figure 2.**
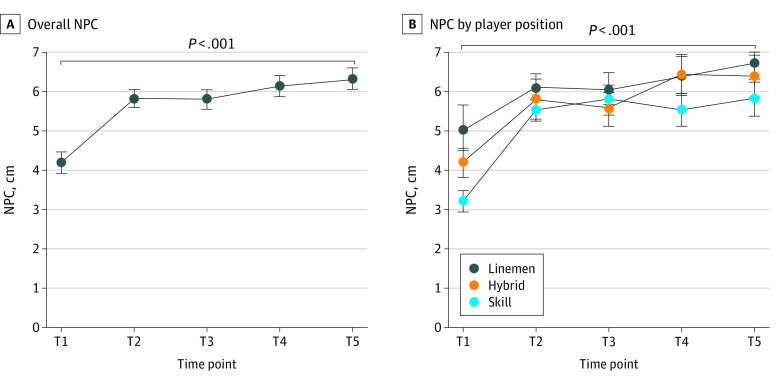
Time-Course Changes in Near Point of Convergence (NPC) Overall and by Player Position Data were collected during the 2021 preseason (July [T1]), after training camp (August [T2]), during the season (September [T3] and October [T4]), and postseason (November or December [T5]). Whiskers indicate standard error of the mean.

There were statistically significant time-course elevations (worsening) in NPC during the season. The NPC distance changes increased sharply after the training camp (T2), continued to worsen toward the end of the season (T4), and peaked at postseason (T5) compared with the preseason baseline (T2: 1.74 cm [95% CI, 1.32-2.16 cm; *P* < .001]; T3: 1.69 cm [95% CI, 1.27-2.11 cm; *P* < .001]; T4: 2.03 cm [95% CI, 1.61-2.45 cm; *P* < .001]; and T5: 2.21 cm [95% CI, 1.80-2.63 cm; *P* < .001]) (Figure 2A).

**Figure 3.**
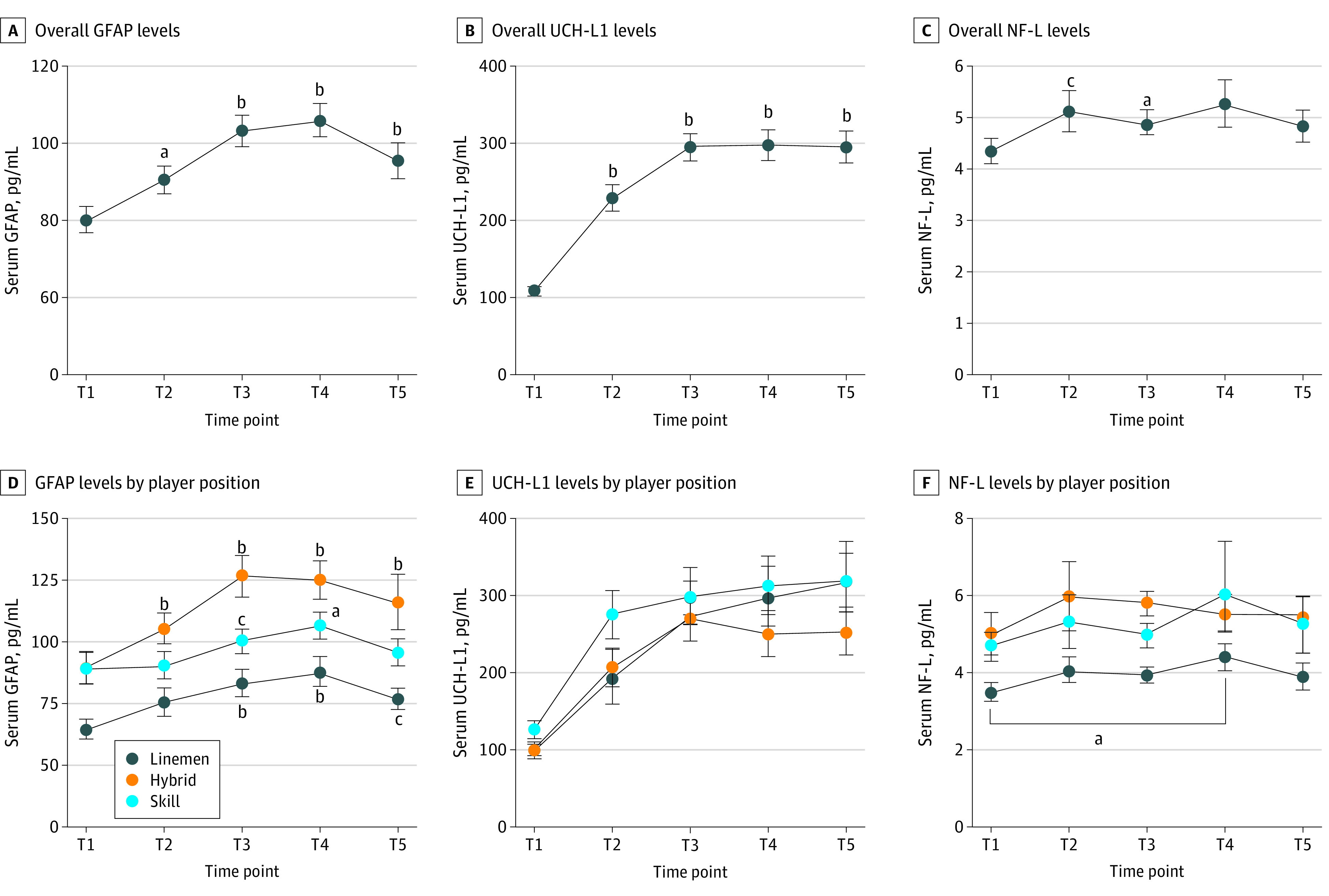
Time-Course Changes in Blood Biomarker Levels Overall and by Player Position Data were collected during the 2021 preseason (July [T1]), after training camp (August [T2]), during the season (September [T3] and October [T4]), and postseason (November or December [T5]). E. *P* < .001 vs preseason for all comparisons. GFAP indicates glial fibrillary acidic protein; NF-L, neurofilament light; and UCH-L1, ubiquitin C-terminal hydrolase-L1. Whiskers indicate standard error of the mean. ^a^*P* < .01 vs preseason. ^b^*P* < .001 vs preseason. ^c^*P* < .05 vs preseason.

All 3 blood biomarkers showed statistically significant time-course changes. Gradual elevations in GFAP level were found as the season progressed, and the level peaked at T4 and declined at T5. At peak, the mean GFAP level increased by 25.6 pg/mL (95% CI, 17.6-33.6 pg/mL) (*P* < .001) (Figure 3A) compared with baseline. Substantial increases in UCH-L1 levels were found earlier in the season, then plateaued thereafter (T3, T4, and T5). At peak (T4), UCH-L1 level increased by 188.5 pg/mL (95% CI, 145.6-231.4 pg/mL; *P* < .001) (Figure 3B) compared with baseline. The time-course change in NF-L levels was modest, such that significant elevations were observed at T2 (0.78 pg/mL; 95% CI, 0.14-1.41 pg/mL; *P* = .011) and T3 (0.55 pg/mL; 95% CI, 0.13-0.99 pg/mL; *P* = .006) but not at T4 and T5 (Figure 3C). eTable 1 in Supplement 1 shows mean values and eTable 2 in Supplement 1 shows changes in outcome variables.

### Association of Players’ Positions With Changes in NPC and Blood Biomarker Levels

There were no statistically significant group differences in mean (SD) head impact exposures during the season among position groups (linemen, 115 [124] hits; hybrid, 112 [93] hits; skill, 74 [115] hits; *P* = .71). This translated into no clear differences in the time-course changes in NPC (Figure 2B) and blood biomarker levels among positions (Figure 3D-F), in which all groups increased at similar degrees. However, the lineman group showed lower levels of GFAP and NF-L levels at various time points compared with the other 2 groups (Figure 3D and E). For instance, compared with the lineman group, the hybrid and skill groups showed 17.4 to 43.2 pg/mL higher mean GFAP levels and 1.1 to 1.9 pg/mL higher mean NF-L levels during the season. The extent of the group differences is detailed in eTable 3 in Supplement 1.

### Associations Between Head Impact Kinematics and Changes in NPC and Blood Biomarker Levels

**Figure 4.**
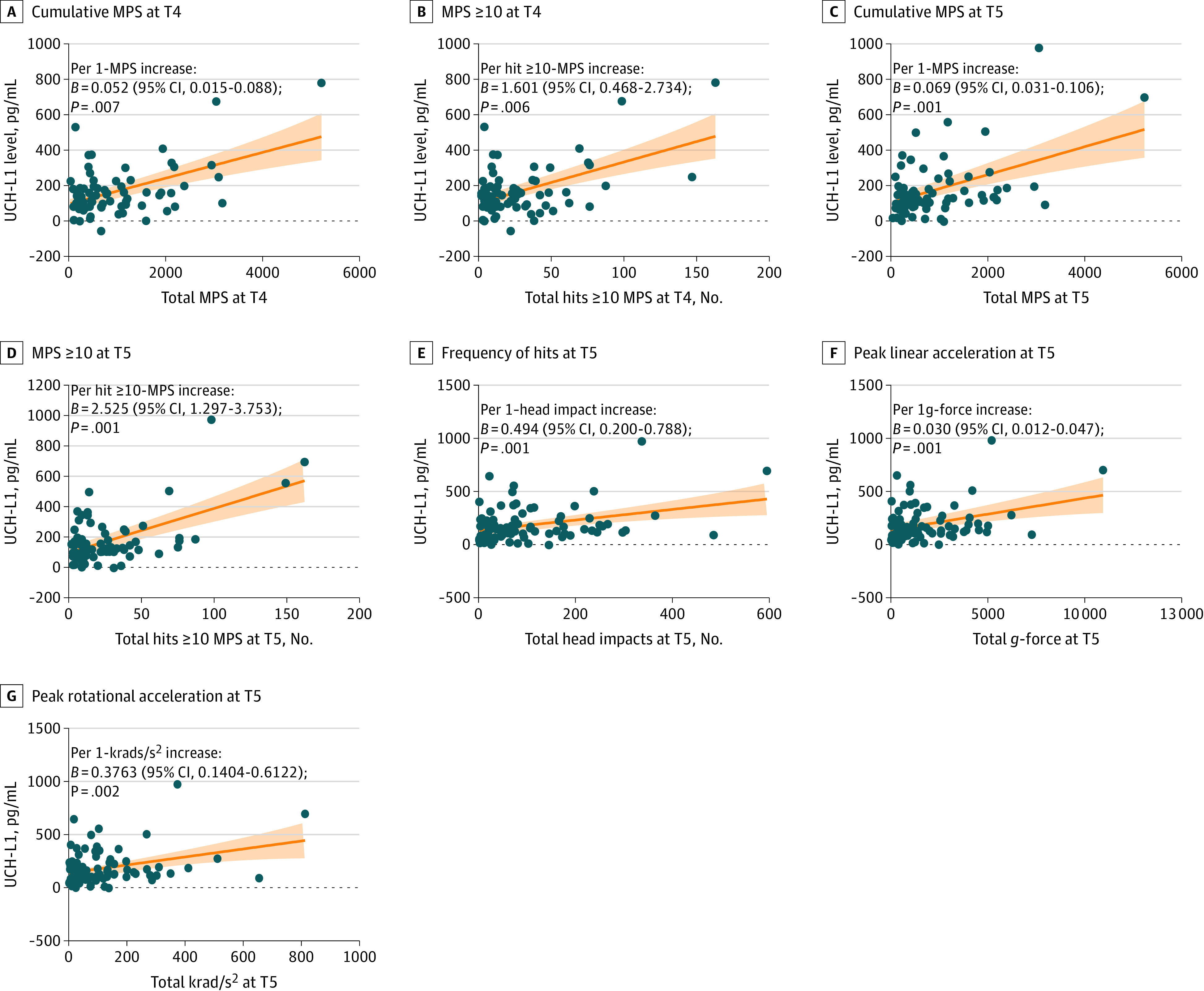
Associations Between Changes in Ubiquitin C-Terminal Hydrolase-L1 (UCH-L1) and Tissue Strain and Kinematic Variables Orange lines are estimated slope based on regression models; shading represents 95% CIs. MPS indicates maximum principal strain; T4, during the season (October 2021); and T5, postseason (November or December 2021).

There were no notable associations of NPC, GFAP, and NF-L with any kinematic variables at any time point. However, the changes in UCH-L1 level were positively correlated with kinematic variables (frequency of hits: *B* = 0.494 [95% CI, 0.200-0.788; *P* = .001]; PLA: *B* = 0.030 [95% CI, 0.012-0.047; *P* = .001]; PRA: *B* = 0.376 [95% CI, 0.140-0.612; *P* = .002]) at postseason (T5). Moreover, both the sum of MPS and an MPS of 10 or greater were significantly correlated with changes in UCH-L1 levels at T4 (cumulative MPS: *B* = 0.052 [95% CI, 0.015-0.088; *P* = .007]; MPS ≥10: *B* = 1.601 [95% CI, 0.468-2.734; *P* = .006]) and T5 (cumulative MPS: *B* = 0.069 [95% CI, 0.031-0.106; *P* = .001]; MPS ≥10: *B* = 2.525 [95% CI, 1.297-3.753; *P* = .001]). Figure 4 shows the visual output and eTable 4 in Supplement 1 shows the statistical output.

## Discussion

The novelty of this study was its assessment of longitudinal multimodal associations between NPC, blood biomarker levels (GFAP, NF-L, and UCH-L1), head impact kinematics (frequency, PLA, and PRA), and an estimated strain measure (MPS). There were 4 primary findings. First, there was a sharp increase in NPC during the training camp; then, NPC gradually increased as the season progressed. Second, all 3 blood biomarkers, particularly GFAP and UCH-L1, showed significant elevations across the season and either peaked or plateaued toward the end of the season. Third, there was a time trend in all markers by player position despite no significant position differences in the degrees of changes in NPC and GFAP, NF-L, and UCH-L1 levels. Lastly, both impact kinematics and MPS were associated with changes in UCH-L1 level later in the season, particularly MPS. Collectively, these data suggest that adolescent football players may accumulate neuronal cellular and functional impairments during a season and that the impact kinematics and degree of brain tissue strain may reflect the extent of subconcussive neural stress.

Contrary to our hypothesis, the degree of increases in the neurological outcomes was independent from players’ positions. There has been a long-standing societal perception that linemen have greater risks for developing neurodegenerative conditions compared with those in other positions. In fact, linemen in all levels (eg, high school,^27^ college,^28,29^ and professional^30^) have been shown to sustain the greatest number of head impacts because of the nature of their task on the field. However, the number of head impacts does not necessarily predict neurological outcomes, such that high-speed skill position players (eg, defensive back, running back) have shown greater cortical strain rates^31^ and higher serum expressions of GFAP, NF-L, and tau at postseason compared with players in hybrid and linemen positions.^32^ This evidence suggests that despite linemen typically incurring a high dose of head impacts, player position may not be indicative of neurological outcomes after a football season.

The role of head impact magnitude was explored through kinematic and strain metrics, in which MPS used linear and rotational acceleration data to estimate degrees of brain tissue deformation. Greater brain strains (MPS) often depend on an impact location (eg, lateral hit), high rotational head acceleration,^33,34^ and level of play (eg, college greater than varsity greater than junior varsity).^35,36^ A study using diffusion tensor imaging in youth football players found that changes in diffusion tensor imaging metrics (eg, fractional anisotropy) at postseason compared with preseason baseline were better explained by strain-based metrics, such as tensile, compressive, and shear strain, than by kinematic-based metrics like PLA and PRA.^37^ One of the reasons why correlations with MPS were unique to UCH-L1 may be that MPS reflects strains and stretching forces at the tissue level instead of cellular components, such as axonal microstructure or glial activations. Since UCH-L1 is abundantly present in all types of neurons and participates in neuronal function and survival,^38^ changes in UCH-L1 may surrogate an injury to diffusive areas of the brain. For example, UCH-L1 levels are elevated by 73% after a football game^39^ and by 200% in an emergency department setting after subconcussive injury.^40^ Furthermore, Joseph et al^17^ reported that UCH-L1 levels acutely increased by 738% in athletes sustaining a high magnitude of head impacts (PLA >95*g*, PRA >3760 rad/s^2^) and mean (SD) UCH-L1 levels increased (144 [56] pg/mL) postseason compared with the preseason baseline, which is in line with our results. Our data expand on these studies by finding that UCH-L1 showed gradual elevations during the first 2 months of the season and plateaued thereafter at 2-fold higher levels than the baseline, and the correlations between UCH-L1 and MPS portend potential brain tissue damage.

The clinical finding of this study is also noteworthy. The NPC displayed chronic and lingering impairments without normalizing to baseline levels even at postseason. Impairment in the oculomotor system has been recognized as a hallmark clinical outcome of concussion and subconcussive head impacts.^14^ For example, NPC was able to distinguish concussed athletes from healthy controls with 73% accuracy.^41^ Nearly half of concussed patients in athletic and military populations showed at least a 2-fold worsening in NPC.^42,43^ As few as 10 acute soccer headings have yielded a 17% to 24% increase in NPC, and the impairment in NPC persisted even after 24 hours.^9,10^ In our study, NPC sharply increased during the training camp, and the elevations lingered throughout the season and peaked at the postseason. These data suggest that when evaluating a suspected concussion case, it may be important to account for 30% to 40% of NPC increase from baseline if the patient is exposed to many subconcussive head impacts prior to a concussion.

### Limitations

This study has several limitations. While this is one of the largest football studies in adolescents, it would have benefited from a more racially and ethnically diverse sample. Given that there are extraneous factors (eg, exercise, heat, and hydration) inherent to field studies, it would be beneficial to investigate chronic head impact effects in more controlled settings, such as the use of a soccer heading model.^13^ Additionally, the data cannot address any long-term (multiyear) effects of subconcussive head impacts, yet we are currently conducting a study with longer-term follow-ups with noncontact controls from various sports.

## Conclusions

Data from this cohort study suggest that adolescent football players experience oculomotor impairments and elevations in blood biomarker levels associated with astrocyte activation and neuronal injury. Changes in serum UCH-L1 levels were associated with the extent of brain tissue strain and kinematic variables, but no longitudinal association was observed between head impact kinematics or strain measures and NPC or GFAP and NF-L levels. Assessment of long-term effects of subconcussive head impacts in adolescent football players will require at least several years of follow-up within longitudinal studies.

## Notes

Supplement 1. **eTable 1.** Group Average Values of Outcomes at Each Time Point **eTable 2.** Changes in Outcome Variables Relative to the Preseason Baseline **eTable 3.** Group Differences in Outcome Variables at Each Time Point **eTable 4.** Associations Between Changes in Blood Biomarkers and Head Impact Kinematics and MPS Across the Season **eFigure.** Study Design

Supplement 2.
Data Sharing Statement
