# Supplementary material for: Longitudinal Associations of Clinical and Biochemical Head Injury Biomarkers With Head Impact Exposure in Adolescent Football Players
Source: JAMA Netw Open. 2023 May 30;6(5):e2316601. doi: 10.1001/jamanetworkopen.2023.16601 (PMC10230318; doi:10.1001/jamanetworkopen.2023.16601)
Supplement: Supplement 1. — eTable 1. Group Average Values of Outcomes at Each Time Point eTable 2. Changes in Outcome Variables Relative to the Preseason Baseline eTable 3. Group Differences in Outcome Variables at Each Time Point eTable 4. Associations Between Changes in Blood Biomarkers and Head Impact Kinematics and MPS Across the Season eFigure. Study Design [file jamanetwopen-e2316601-s001.pdf]

Supplementary Online Content

Zuidema TR, Bazarian JJ, Kercher KA, et al. Longitudinal associations of clinical and biochemical head injury biomarkers with head impact exposure in adolescent football players. *JAMA Netw Open*. 2023;6(5):e2316601.
doi:10.1001/jamanetworkopen.2023.16601

eTable 1. Group Average Values of Outcomes at Each Time Point
eTable 2. Changes in Outcome Variables Relative to the Preseason Baseline
eTable 3. Group Differences in Outcome Variables at Each Time Point
eTable 4. Associations Between Changes in Blood Biomarkers and Head Impact Kinematics and MPS Across the Season
eFigure. Study Design

This supplementary material has been provided by the authors to give readers additional information about their work.

© 2023 Zuidema TR et al. *JAMA Network Open*.

eTable 1: Group average values of outcomes at each time point						
		T1 Preseason (July)	T2 Post-camp (August)	T3 In-season (September)	T4 In-Season (October)	T5 Postseason (November)
NPC (cm)	Overall	4.3 ± 2.6	5.9 ± 1.9	5.9 ± 2.2	6.2 ± 2.7	6.3 ± 2.5
	Lineman	5.8 ± 3.4	6.1 ± 1.7	6.1 ± 2.3	6.4 ± 2.6	6.8 ± 2.4
	Hybrid	4.0 ± 2.0	5.8 ± 2.6	5.5 ± 2.3	6.4 ± 2.7	6.4 ± 2.8
	Skill	3.2 ± 1.4	5.5 ± 1.3	5.8 ± 2.0	5.6 ± 2.2	5.8 ± 2.2
GFAP (pg/mL)	Overall	80.5 ± 32.6	90.8 ± 32.2	103.4 ± 38.5	106.1 ± 39.2	95.7 ± 43.5
	Lineman	65.0 ± 21.8	75.8 ± 27.8	83.6 ± 28.5	87.9 ± 34.3	76.6 ± 24.4
	Hybrid	89.4 ± 35.1	105.4 ± 33.6	126.7 ± 43.4	125.1 ± 43.0	116.2 ± 60.6
	Skill	89.4 ± 34.8	90.6 ± 28.4	101.0 ± 28.8	106.6 ± 31.0	95.7 ± 26.8
UCH-L1 (pg/mL)	Overall	108.8 ± 53.1	224.9 ± 157.3	289.9 ± 155.9	288.1 ± 178.1	299.3 ± 192.4
	Lineman	100.1 ± 56.9	196.4 ± 180.7	300.9 ± 192.7	300.1 ± 197.0	326.6 ± 236.8
	Hybrid	100.7 ± 42.1	207.6 ± 129.9	271.8 ± 153.9	251.7 ± 149.9	253.0 ± 155.2
	Skill	127.7 ± 56.2	277.3 ± 152.0	298.1 ± 107.1	314.6 ± 185.7	320.7 ± 171.6
NF-L (pg/mL)	Overall	4.4 ± 2.3	5.1 ± 3.7	4.9 ± 2.3	5.3 ± 4.3	4.8 ± 2.9
	Lineman	3.5 ± 1.5	4.1 ± 1.7	4.0 ± 1.3	4.4 ± 1.6	3.9 ± 1.7
	Hybrid	5.0 ± 3.0	6.0 ± 4.8	5.8 ± 2.9	5.5 ± 2.4	5.5 ± 2.7
	Skill	4.7 ± 2.1	5.3 ± 3.5	5.1 ± 2.1	6.0 ± 7.0	5.3 ± 3.9
Note: NPC, near point of convergence. GFAP, glial fibrillary acidic protein. UCH-L1, Ubiquitin carboxy-terminal hydrolase L1. NF-L, neurofilament light.						

© 2023 Zuidema TR et al. *JAMA Network Open*.

eTable 2: Changes in outcome variables relative to the preseason baseline					
		T2 Post-camp (August)	T3 In-season (September)	T4 In-Season (October)	T5 Postseason (November)
NPC (cm)	Change from T1	1.6 (1.0, 2.2)	1.6 (1.0, 2.2)	1.9 (1.4, 2.5)	2.1 (1.5, 2.7)
	p-value	< 0.0001	< 0.0001	< 0.0001	< 0.0001
GFAP (pg/mL)	Change from T1	10.3 (4.1, 16.5)	22.9 (15.9, 29.9)	25.6 (17.6, 33.6)	15.2 (6.9, 23.6)
	p-value	0.0003	<0.0001	<0.0001	<0.0001
UCH-L1 (pg/mL)	Change from T1	120.2 (84.7, 155.6)	185.6 (147.5, 223.7)	188.5 (145.6, 231.4)	186.0 (142.4, 229.7)
	p-value	<0.0001	<0.0001	<0.0001	<0.0001
NF-L (pg/mL)	Change from T1	0.78 (0.14, 1.41)	0.56 (0.13, 0.99)	0.93 (0.09, 1.96)	0.49 (0.10, 1.07)
	p-value	0.0108	0.0060	0.0836	0.1301
Note: Values are expressed as difference (95% confidence interval). NPC, near point of convergence. GFAP, glial fibrillary acidic protein. UCH-L1, Ubiquitin carboxy-terminal hydrolase L1. NF-L, neurofilament light.					

© 2023 Zuidema TR et al. *JAMA Network Open*.

eTable 3: Group differences in outcome variables at each time point. Lineman as a reference group

		T1 Preseason (July)	T2 Post-camp (August)	T3 In-season (September)	T4 In-Season (October)	T5 Postseason (November)
NPC (cm)	Hybrid vs. Lineman	-0.9 (-2.5, 0.7)	-0.3 (-1.7, 1.0)	0.5 (-1.9, 0.8)	0 (-1.6, 1.6)	-0.4 (-1.9, 1.2)
	Skill vs. Lineman	-1.8 (-3.3, -0.4)*	-0.6 (-1.5, 0.3)	-0.3 (-1.6, 1.0)	-0.9 (-2.3, 0.6)	-0.9 (-2.3, 0.5)
GFAP (pg/mL)	Hybrid vs. Lineman	24.4 (7.1, 41.6)**	29.6 (11.1, 48.2)**	43.2 (21.4, 65.0)***	37.2 (13.8, 60.5)**	39.5 (11.7, 67.4)**
	Skill vs. Lineman	24.4 (7.0, 41.8)**	14.9 (-2.6, 32.3)	17.4 (0.1, 34.8)*	-18.6 (-1.0, 38.3)	19.1 (3.63, 34.5)*
UCH-L1 (pg/mL)	Hybrid vs. Lineman	0.6 (-29.7, 30.9)	11.2 (-87.8, 110.3)	-29.2 (-137.3, 79.0)	-48.4 (-159.2, 62.3)	-73.6 (-198.2, 51.0)
	Skill vs. Lineman	27.5 (-7.6, 62.7)	80.9 (27.6, 189.3)	-2.8 (-102.0, 96.4)	14.5 (-108.9, 137.9)	-5.9 (-137.1, 125.3)
NF-L (pg/mL)	Hybrid vs. Lineman	1.5 (0.1, 2.9)*	1.9 (-0.3, 4.1)	1.9 (0.5, 3.2)**	1.1 (-0.1, 2.4)	1.6 (0.2, 2.9)*
	Skill vs. Lineman	1.2 (0.1, 2.2)*	1.2 (-0.5, 3.0)	1.1 (0.04, 2.2)*	1.6 (-1.6, 4.8)	1.3 (-0.5, 3.2)

Note: Values are expressed as difference (95% confidence interval). NPC, near point of convergence. GFAP, glial fibrillary acidic protein. UCH-L1, Ubiquitin carboxy-terminal hydrolase L1. NF-L, neurofilament light. Shaded cells indicate significant difference compared to the lineman group. *p<0.05, **p<0.01, ***p<0.001.

© 2023 Zuidema TR et al. *JAMA Network Open*.

eTable 4: Associations between changes in blood biomarkers and head impact kinematics and MPS across the season.					
		T2 Post-Camp (August)	T3 In-Season (September)	T4 In-Season (October)	T5 Post-Season (November)
Frequency	GFAP	- 0.004 (-0.101, 0.093) *p* = 0.929	0.025 (-0.040, 0.089) *p* = 0.452	0.057 (-0.001, 0.114) *p* = 0.053	0.027 (-0.032, 0.086) *p* = 0.365
	NF-L	- 0.004 (-0.014, 0.005) *p* = 0.368	-0.001 (-0.005, 0.003) *p* = 0.693	-0.001 (-0.009, 0.007) *p* = 0.790	-0.000 (-0.004, 0.004) *p* = 0.881
	UCH-L1	0.092 (-0.457, 0.641) *p* = 0.739	0.233 (-0.116, 0.581) *p* = 0.189	0.369 (0.062, 0.656) *p* = 0.019	0.494 (0.200, 0.788) *p* = 0.001
PLA	GFAP	-0.000 (-0.006, 0.006) *p* = 0.900	0.002 (-0.002, 0.005) *p* = 0.399	0.003 (0.000, 0.007) *p* = 0.045	0.002 (-0.002, 0.005) *p* = 0.290
	NF-L	-0.001 (-0.001, 0.000) *p* = 0.440	-0.003 (-0.000, 0.000) *p* = 0.798	0.000 (-0.000, 0.000) *p* = 0.850	0.000 (-0.000, 0.000) *p* = 0.968
	UCH-L1	0.006 (-0.027, 0.040) *p* = 0.705	0.012 (-0.008, 0.033) *p* = 0.243	0.022 (0.004, 0.039) *p* = 0.016	0.030 (0.012, 0.047) *p* = 0.001
PRA	GFAP	0.000 (-0.000, 0.000) *p* = 0.948	0.000 (-0.000, 0.000) *p* = 0.395	0.000 (-0.000, 0.000) *p* = 0.078	0.000 (-0.000, 0.000) *p* = 0.209
	NF-L	0.000 (-0.000, 0.000) *p* = 0.319	0.000 (-0.000, 0.000) *p* = 0.596	0.000 (-0.000, 0.000) *p* = 0.724	0.000 (-0.000, 0.000) *p* = 0.978

© 2023 Zuidema TR et al. *JAMA Network Open*.

	UCH-L1	0.000 (-0.000, 0.001) *p* = 0.597	0.000 (-0.000, 0.000) *p* = 0.460	0.000 (-0.000, 0.001) *p* = 0.018	0.000 (-0.000, 0.001) *p* = 0.002
MPS Sum	GFAP	0.000 (-0.011, 0.010) *p* = 0.934	0.002 (-0.006, 0.010) *p* = 0.573	0.006 (-0.001, 0.013) *p* = 0.106	0.005 (-0.003, 0.012) *p* = 0.236
	NF-L	-0.001 (-0.002, 0.001) *p* = 0.315	0.000 (-0.001, 0.000) *p* = 0.786	0.000 (-0.001, 0.001) *p* = 0.737	0.000 (-0.001, 0.001) *p* = 0.938
	UCH-L1	0.021 (-0.042, 0.083) *p* = 0.515	0.020 (-0.022, 0.062) *p* = 0.346	0.052 (0.015, 0.088) *p* = 0.007	0.069 (0.031, 0.106) *p* < 0.001
# hits > 10 MPS	GFAP	-0.021 (-0.327, 0.286) *p* = 0.895	0.046 (-0.179, 0.270) *p* = 0.686	0.183 (-0.041, 0.408) *p* = 0.108	0.211 (-0.041, 0.464) *p* = 0.100
	NF-L	-0.010 (-0.042, 0.021) *p* = 0.515	0.000 (-0.014, 0.013) *p* = 0.949	-0.005 (-0.034, 0.024) *p* = 0.747	0.006 (-0.012, 0.024) *p* = 0.512
	UCH-L1	0.544 (-1.222, 2.309) *p* = 0.542	0.473 (-0.736, 1.683) *p* = 0.439	1.601 (0.468, 2.734) *p* = 0.006	2.525 (1.297, 3.753) *p* < 0.0001

Note: Values are expressed as associations (95% confidence interval). Frequency, number of head impacts. PLA, peak linear acceleration. PRA, peak rotational acceleration. MPS, maximum principal strain. GFAP, glial fibrillary acidic protein. NF-L, neurofilament light. UCH-L1, Ubiquitin carboxy-terminal hydrolase L1. Dark cells indicate a significant association at a level below p=0.01. Light cells indicate an association within the range of p=0.05–0.01.

© 2023 Zuidema TR et al. *JAMA Network Open*.

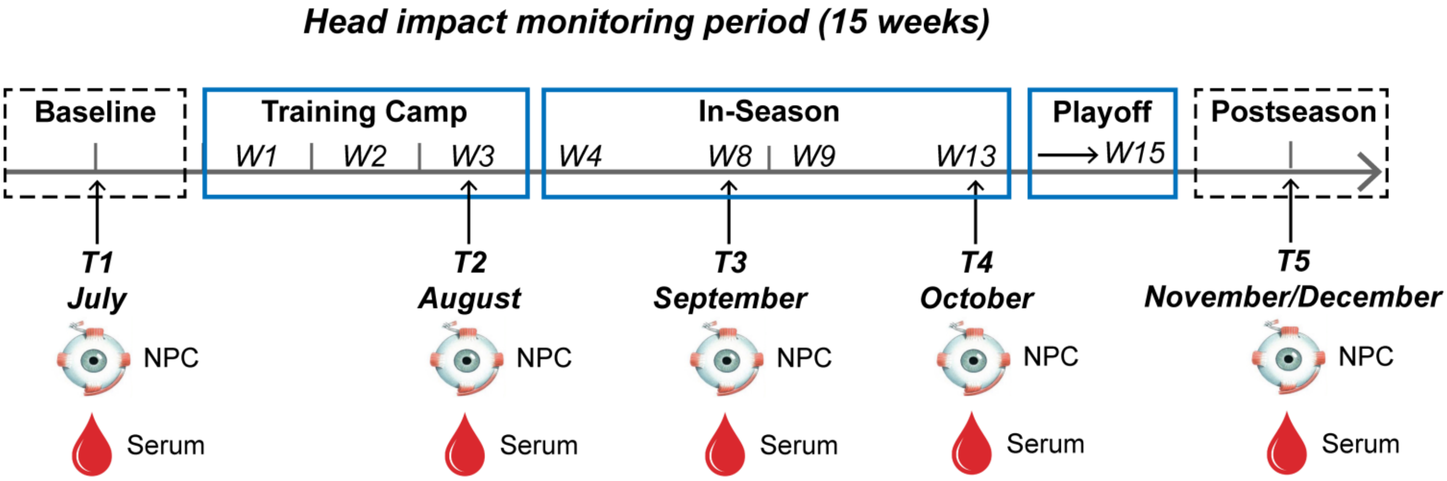

eFigure: Study design

© 2023 Zuidema TR et al. *JAMA Network Open*.
