# Supplementary material for: Longitudinal Associations of Clinical and Biochemical Head Injury Biomarkers With Head Impact Exposure in Adolescent Football Players
Source: JAMA Netw Open. 2023 May 30;6(5):e2316601. doi: 10.1001/jamanetworkopen.2023.16601 (PMC10230318; doi:10.1001/jamanetworkopen.2023.16601)
Supplement: Supplement 2. — Data Sharing Statement [file jamanetwopen-e2316601-s002.pdf]

Data Sharing Statement

Zuidema. Longitudinal Associations of Clinical and Biochemical Head Injury Biomarkers With Head Impact Exposure in Adolescent Football Players. *JAMA Netw Open*. Published May 30, 2023. doi:10.1001/jamanetworkopen.2023.16601

Data
Data available: No

Additional Information
Explanation for why data not available: This is part of an ongoing longitudinal study. When the longitudinal tracking is fully executed, we will deposit dataset in public repository (FITBIR).
